# Effects of Drought-Tolerant *Ea-DREB2B* Transgenic Sugarcane on Bacterial Communities in Soil

**DOI:** 10.3389/fmicb.2020.00704

**Published:** 2020-05-05

**Authors:** Xiaowen Zhao, Yuke Jiang, Qi Liu, Huichun Yang, Ziting Wang, Muqing Zhang

**Affiliations:** ^1^Guangxi Key Laboratory of Sugarcane Biology, Nanning, China; ^2^State Key Laboratory for Conservation and Utilization of Subtropical Agro-bioresources, Guangxi University, Nanning, China; ^3^College of Agronomy, Guangxi University, Nanning, China

**Keywords:** drought-tolerant, *Ea-DREB2B*, sugarcane, bacterial community, environmental factor

## Abstract

Drought is a major abiotic stress affecting plant growth and development. Sugarcane, a sugar crop planted in warmer climate, suffers dramatically from drought stress. Bacterial communities colonizing the rhizosphere, where roots sense drought stress initially, have been well studied for their beneficial effects on plant growth and health. The *Ea-DREB2B* gene cloned from the sugarcane, *Saccharum arundinaceum*, belongs to the DREB2 subgroup of the DREB gene family, which is involved in drought response regulation. Here, we present a detailed characterization of the rhizoplane, rhizosphere, and bulk soil bacterial communities determined using a high-sequencing approach with the transgenic (TG) sugarcane variety GN18 harboring the drought-tolerant *Ea-DREB2B* gene and its isogenic wild-type (WT) variety FN95-1702 under the same environmental conditions. In addition, the total carbon (TC), total nitrogen (TN), and total phosphorus (TP) contents in each soil area were compared to explore the relationship between bacterial alteration in the TG and WT plants and environmental factors (TC, TN, TP, C:N, C:P, and N:P). Our results showed that the bacterial communities in the rhizosphere and rhizoplane of TG sugarcane were more similar and perfectly correlated with the environmental factors than those of the WT. This suggested that the bacterial communities of the TG plants were altered in response to the changes in root exudates. The results of our study suggest that the change in soil environment caused by transgenic sugarcane alters soil bacterial communities.

## Introduction

Plants are intimately intertwined with the microbial communities living in and around them (Naylor et al., 2017). The rhizosphere is a small compartment of the soil that is adjacent to and directly affected by the plant roots, and it has long been regarded as one of the most important interfaces for life on Earth. The rhizoplane is the root surface that forms the interface between the plant root and rhizosphere soil (Ding et al., 2019). There is a strong relationship between plant and microbiomes colonized in the rhizosphere. Genetically modified (GM) plants with stress-tolerant ability are prevalent worldwide. Considering the contribution of microbial–plant interactions for plant growth and development, numerous studies have focused on the influence of GM stress-resistant plants on soil- and root-associated bacterial communities (Dunfield and Germida, 2004; Sohn et al., 2016; Ibarra et al., 2020). Some studies also suggest that the modification of certain genes in plants can influence the associated bacterial communities, resulting in a change in the rhizosphere compared with that of the wild-type (WT) plant communities (Brusetti et al., 2005, 2008). Therefore, it is important to assess the specific effect of each GM plant on the soil environment and community.

Drought stress represents one of the most significant obstacles to global crop production and is expected to increase in severity and frequency in the future (Xu L. et al., 2018). As drought severely stunts plant growth and development, several studies have focused on strategies for improving drought resistance from a global perspective. Many genes that play a role in plant responses to drought have been identified, and some of these have been shown to be effective in improving drought tolerance by genetic engineering (Zolla et al., 2013). Sugarcane, an important source of sugar and ethanol, is a relatively high water-demanding crop and its growth is highly sensitive to water deficits (Ferreira et al., 2017). Genetic engineering has been applied in the enhancement of the drought resistance of sugarcane (Ramiro et al., 2016). Genes encoding the dehydration-responsive element-binding (DREB) transcription factors identified in *Arabidopsis thaliana* have been reported to enhance drought resistance in transgenic (TG) plants (Mizoi et al., 2012). *Ea-DREB2B*, cloned from the hardy sugarcane *Saccharum arundinaceum* is a member of the DREB2 family, which is a subfamily of DREB that regulates the expression of several stress-inducible genes and plays a critical role in enhancing the tolerance of plants to drought and salinity (Lata and Prasad, 2011; Augustine et al., 2015). It has been reported that the drought resistance of sugarcane modified by *Ea-DREB2B* was significantly enhanced as compared to that of non-transgenic sugarcane (Xu S. et al., 2018). CBF/DREB regulon is one of the activated regulons of the abscisic acid (ABA)-independent pathway (Saibo et al., 2009). ABA is a plant growth regulator and stress hormone that induces leaf stomata closure to reduce water loss *via* transpiration and decreases the photosynthetic rate to improve the efficiency of water usage by plants (Agarwal et al., 2006). The expression of *SlDREB3* in tomatoes affects several ABA-associated processes by reducing the ABA levels and responses, thereby leading to higher photosynthesis (Upadhyay et al., 2017). The root-associated bacteria are sensitive to even small changes in the pattern of compounds in the rhizosphere (Persello-Cartieaux et al., 2003), which may be altered by genetic modifications for enhancing or conferring specific traits. Although numerous studies have confirmed that DREB2s contribute greatly to the enhanced drought and salinity tolerance of a plant (Chen et al., 2009; Matsukura et al., 2010; Li et al., 2017), few studies have focused on the bacterial communities in the soil of plants modified by DREB2s (Chun-miao et al., 2015). Therefore, it is important to evaluate the influence of plants modified with DREB2 genes on hormone processes that could influence the root-associated bacterial communities.

In this milieu, the main objective of this study was to investigate the effects exerted by the TG sugarcane modified using the *Ea-DREB2B* gene on the root-associated layers of the soil and the bulk soil bacterial communities. The specific aims were to (1) determine the variation in the diversity and composition of TG bacterial communities in the rhizoplane, rhizosphere, and bulk soil compared with those of the non-transgenic WT communities; (2) explore the relationship between the alteration in TG bacterial communities and environmental factors, including total carbon (TC), total nitrogen (TN), and total phosphorus (TP) contents and the C:N, C:P, and N:P ratios; and (3) determine any correlations among the bacterial communities of the rhizoplane, rhizosphere, and bulk soil of TG sugarcane. Our study will provide general insights into the potential effects of genetic modifications on key traits to improve crop production and stress tolerance in a broader ecosystem context and, thus, offer guidance for the development and monitoring of new GM varieties.

## Materials and Methods

### Plants and Field Experiment Design

This study was performed in the forage breeding ground in Quli, Fusui, Chongzuo, China (between 107°31′ and 108°06′E, and 22°17′ and 22°57′ N) of Guangxi University in the summer of 2018. The average annual temperature was 21.3°C. The lowest temperature in the past year was −0.6°C and the highest temperature was 39.5°C. The total annual radiation was 108.4 kcal/cm, with 1,693 h of annual average sunshine, and the frost-free period was up to 346 days. The annual precipitation in the whole region was 1,050–1,300 mm. It was windy and dry in the winter and spring, and rainy and humid in the summer and autumn (Supplementary Figure S1). Fields cultivated over the long term with sugarcane had the following properties: lateritic red earth, pH of 5.15, 19.47 g/kg organic matter, 0.84 g/kg TN, 2.98 g/kg TP, 7.11 g/kg total potassium, 136 mg/kg alkaline-hydrolyzed nitrogen, 83 mg/kg available phosphorus, and 77.1 mg/kg available potassium. GN18 is a TG variety that was derived from FN95-1702 as the acceptor parent material using the inducible promoter RD28A and a gene gun for overexpression of the *Ea-DREB2B* gene to confer greater drought resistance (Xu S. et al., 2018). The resistance of GN18 under drought stress and the ability to recover after rehydration were confirmed to be stronger than those of the acceptor parental material FN95-1702 (Xu S. et al., 2018). The experiment consisted of a random block design with six blocks, with each block covering an area of 30 m × 4.2 m. Each block contained both sugarcane varieties (three lines for each plant). The distance between two varieties was 2.1 m and the distance between two sugarcanes was 30 cm in each block; each line was planted with 46 sugarcanes.

### Soil Sample Collection and Physicochemical Analysis

Soil samples were collected in the late jointing stage on November 18, 2018. Bulk soil was taken adjacent to the excavated sugarcane (20 cm from where the stalk had been) from 0- to 20-cm depth using a standard soil corer. Each sampling site consisted of five subsamples collected between two lines of sugarcane (Wang et al., 2016). Three of five points were selected to dig out sugarcane roots. The rhizospheric compartment was separated by thoroughly vortexing the roots for 20 s and collecting the resulting soil precipitation in PowerBead tubes. The rhizoplane compartment was derived from the root surface, which was removed by sonication for 5 min (Edwards et al., 2015). Seventy-two sugarcane roots were collected altogether from six blocks. Each soil sample consists of soil collected from 12 sugarcane roots which were excavated from two blocks through random selection in six blocks. Each composite soil sample was homogenized and stored at −80°C for less than 24 h before DNA extraction. After DNA extraction, the soil samples were air-dried and passed through a 2-mm sieve before measuring the TC, TN, and TP contents. Each soil sample was set up as three replicates with 0.5 g of each sample. The TC (Batjes, 1996) and TP (Sommers and Nelson, 1972) contents were determined as described previously, and the TN content was determined using the Kjeldahl method (Bremner and Tabatabai, 1972).

### DNA Extraction and Sequencing

The total DNA was extracted from 1 g of each soil sample of three biological replicates, which was replicated three times using the E.Z.N. A soil DNA Kit (Omega Bio-Tek, Inc., Norcross, GA, United States) following the manufacturer’s instructions. The concentration and purity of the total DNA were measured using NanoDrop 2000 (Thermo Fisher Scientific, Wilmington, DE, United States). Primers F338 (5′-ACT CCT ACG GGA GGC AGC A-3′) and R806 (5′-GGA CTA CHV GGG TWT CTA AT-3′) targeting the V4 region of the 16S rRNA gene were used for polymerase chain reaction (PCR) (Peiffer et al., 2013). This primer set provides a comprehensive coverage with the highest taxonomical accuracy for bacterial sequences. The reverse primer also contained a 6-bp error-correcting barcode unique to each sample. PCR comprised 25 μl of 2 × Premix Taq (Takara Biotechnology, Dalian Co. Ltd., China), 1 μl of each primer (10 mM), and 3 μl DNA template (20 ng/μl) in a total volume of 50 μl. The reaction conditions were: initial denaturation for 5 min at 94°C; followed by 30 cycles of denaturation at 94°C for 30 s, annealing at 52°C for 30 s, and extension at 72°C for 30 s; and a final elongation at 72°C for 10 min. The quantitative PCR was carried out using BioRad S1000 (Bio-Rad Laboratory, CA, United States). Each PCR product was subjected to sequencing by Magigene Technology (Guangzhou, China) using the Illumina HiSeq 2500 platform. FLASH software was used to merge pairs of reads from the original DNA fragments (Magoč and Salzberg, 2011). Further sequence analysis was performed using USEARCH v5.2.32 and was clustered using Unoise3. The quantitative insights into microbial ecology (QIIME) pipeline software was used to select 16S rRNA operational taxonomic units from the combined reads (Edgar, 2010). The 16S rRNA gene sequences obtained in this study have been deposited in the NCBI sequence read archive (SRA) database with accession number SRP238824.

### Statistical and Bioinformatics Analysis

Alpha diversity was estimated using the Chao1 richness index and Shannon diversity index. Correlations between the alpha diversity and environmental factors were determined using the “corrplot” package (Wei et al., 2017) in the R v3.6.3. environment. Beta diversity, using the principal coordinates analysis (PCoA), was estimated using the Bray–Curtis distance matrix. Furthermore, we used the Mantel test to study the relationship between beta diversity and environmental factors. The Mantel test, PCoA, and distance-based redundancy analysis (dbRDA) were performed using “vegan” packages in R v3.6.3 (Oksanen et al., 2013). The relative abundance of bacterial communities was evaluated in “alluvial” and “ggplot” packages in R v3.6.3 (Wickham and Wickham, 2007), which showed the changing tendency of bacterial populations in each compartment, and the Simper function was used to make pairwise comparisons of population composition. Functional Annotation of Prokaryotic Taxa (FAPROTAX), a database that estimates the metabolism or other ecologically related functions of prokaryotes by extrapolating their functions, was used to predict the functions of rhizosphere bacterial communities under TG and WT intercropping patterns (Louca et al., 2016).

Networks were constructed for root-associated area and bulk soil communities based on operational taxonomic unit (OTU) relative abundances, resulting in two networks. Covariations were measured across nine biological replicates to create each network. Only OTUs detected in five out of nine replicate samples were used for network construction. Random matrix theory (RMT) was used to automatically identify the appropriate similarity threshold (St) prior to network construction; St defines the minimal strength of the connections between each pair of nodes (Deng et al., 2012). Global network properties were characterized according to Zhou et al. (2013). All analyses were performed using the molecular ecological network analyses (MENA) pipeline^1^ and networks were graphed using Cytoscape 2.8.2 (Shannon et al., 2003). We characterized the modularity for each network created in this study. A module is a group of nodes (i.e., OTUs) that are highly connected within the group with few connections outside the group (Newman, 2006). In this study, modules were detected using the greedy modularity optimization method (Deng et al., 2012). Modularity (*M*) is an index measuring the extent to which a network is divided into modules, and we used *M* > 0.4 as the threshold to define modular structures. The connectivity of each node was determined based on its within-module connectivity (*Z*_*i*_) and among-module connectivity (*P*_*i*_), which were then used to classify the nodes based on the topological roles they play in the network (Guimera and Nunes Amaral, 2005). Node topologies are organized into four categories: module hubs (highly connected nodes within modules, *Z*_*i*_ > 2.5), network hubs (highly connected nodes within an entire network, *Z*_*i*_ > 2.5 and *P*_*i*_ > 0.62), connectors (nodes that connect modules, *P*_*i*_ > 0.62), and peripherals (nodes connected in modules with few outside connections, *Z*_*i*_ < 2.5 and *P*_*i*_ < 0.62) (Deng et al., 2012).

## Results

### Soil Chemical Properties, Bacterial Alpha, and Beta Diversity

**FIGURE 1:**
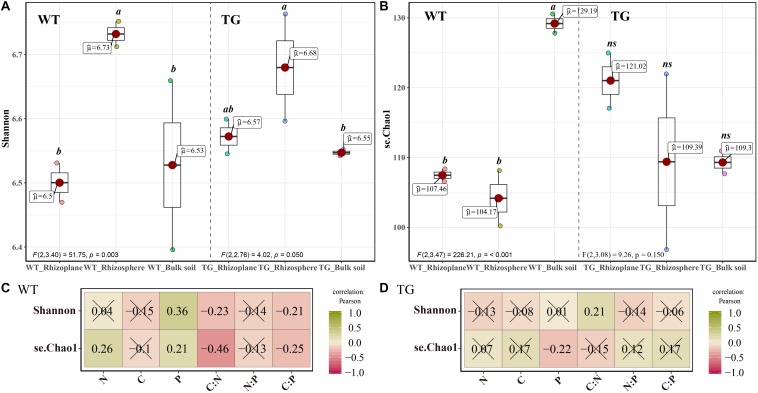
**(A,B)** Bacterial alpha-diversity measurements of represented by Chao 1 Richness and Shannon Diversity in each area and cultivar. **(C,D)** The correlation between bacterial alpha-diversity and environmental factors using Pearson analysis.

The TN content and TC/TN ration were significantly different between the two breeds. Furthermore, significant differences existed in the TC, TN, TC/TN, and TN/TP among the different soil layers. Considering the two influential factors together, there were extremely significant differences in TN and TP (Table 1). The alpha diversity indices, represented by the Chao1 richness and Shannon diversity indices, for the soil bacterial communities were significantly different between the TG and WT plants. However, no significant difference was observed between the rhizoplane and rhizosphere bacterial alpha diversity according to either index for the TG groups, whereas a difference was observed for the WT samples. Moreover, both indices demonstrated a certain degree of increase in the bacterial diversity of TG rhizocompartments compared with those of the WT (Figures 1A,B). Pearson’s correlation analysis indicated that the Shannon diversity index of the WT plants was negatively correlated with the C/N ratio (*P* < 0.05), whereas that of the TG plants was positively correlated with the C/N ratio (*P* < 0.05). Furthermore, the Chao1 index of the WT group was positively correlated with the TP content (*P* < 0.05) and negatively correlated with the TC content (*P* < 0.05), whereas that of the TG group showed the opposite relationships (Figures 1C,D).

**TABLE 1:** Soil chemical properties and the ratio between them according to different compartments.

**Line**	**Root compartment**	**TC (g⋅kg^–1^)**	**TN (g⋅kg^–1^)**	**TP (mg⋅kg^1^)**	**TC/TN**	**TN/TP**	**TC/TP**
WT	Rhizoplane	14.03 ± 2.62ab	1.21 ± 0.20a	4.86 ± 1.59a	11.51 ± 0.58b	0.28 ± 0.15b	3.26 ± 1.73a
	Rhizosphere	11.43 ± 1.88ab	0.88 ± 0.02a	10.82 ± 0.66c	12.95 ± 2.37bc	0.08 ± 0.00a	1.07 ± 0.24a
	Bulk soil	11.88 ± 1.95ab	0.74 ± 0.02a	3.89 ± 0.69a	15.95 ± 2.29c	0.20 ± 0.04ab	3.18 ± 1.04a
TG	Rhizoplane	15.75 ± 1.80b	2.97 ± 0.56b	10.42 ± 0.15bc	5.35 ± 0.42a	0.29 ± 0.06b	1.51 ± 0.19a
	Rhizosphere	13.52 ± 0.58ab	1.21 ± 0.08a	5.77 ± 0.65ab	11.22 ± 0.25b	0.21 ± 0.04ab	2.37 ± 0.37a
	Bulk soil	9.73 ± 0.91a	0.67 ± 0.05a	10.45 ± 3.75bc	14.48 ± 1.42bc	0.07 ± 0.02a	1.04 ± 0.44a
Line		0.518	<0.001***	0.014*	<0.001***	0.938	0.057
Root compartment		0.006**	<0.001***	0.542	<0.001***	0.005**	0.432
Line × Root compartment		0.110	<0.001***	<0.001***	0.032*	0.026*	0.009**

a TC, total carbon; TN, total nitrogen; TP, total phosphate. b Different letters indicate significant differences (ANOVA, P <0.05, Turkey’s HSD post-hoc analysis) among root compartment. c *0.01 < *P* value < 0.05; **P value < 0.01; ***P value < 0.001.

**FIGURE 2:**
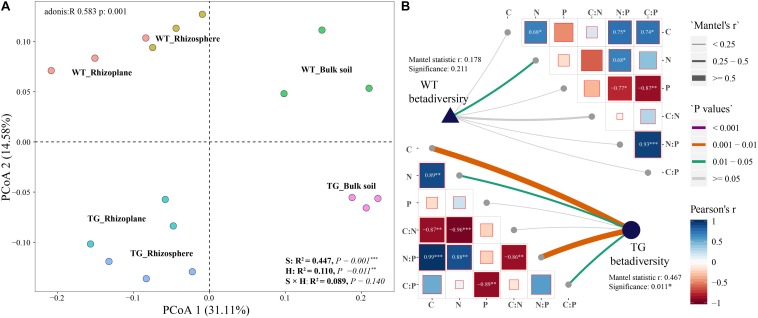
**(A)** Principal coordinate analyses (PCoAs) using Bray distance metric indicates that the largest separation between bacterial communities is spatial distribution of three areas (PCoA.1) and the second largest source of variation is cultivar (PCoA.2). **(B)** Correlation between environmental factors and correlation between bacterial beta-diversity and environmental factors in two sugarcane cultivars using Mantel test.

The PCoA of the Bray distance was performed to investigate and visualize the patterns of separation among the three zones of the two sugarcanes. An obvious overlap was observed in the rhizosphere and rhizoplane areas in TG. In contrast, no apparent intersection was detected between the rhizoplane and rhizosphere of WT plants. In addition, the distance between the bulk soil bacteria community and the rhizosphere of TG plants was further than that of the WT (Figure 2A). The Mantel test further showed that the correlation between environmental factors in TG plants was greater than that in WT. The bacterial beta diversity of TG plants was positively correlated with the TC and TN contents, and the N/P and C/P ratios, respectively, and the correlations between TG bacterial beta diversity and the TC content and N/P ratio were relatively stronger than the others. However, the beta diversity of WT bacterial communities was only positively correlated with the TN content (Figure 2B).

### Influence of the Genetic Modification on the Bacterial Community Composition

**FIGURE 3:**
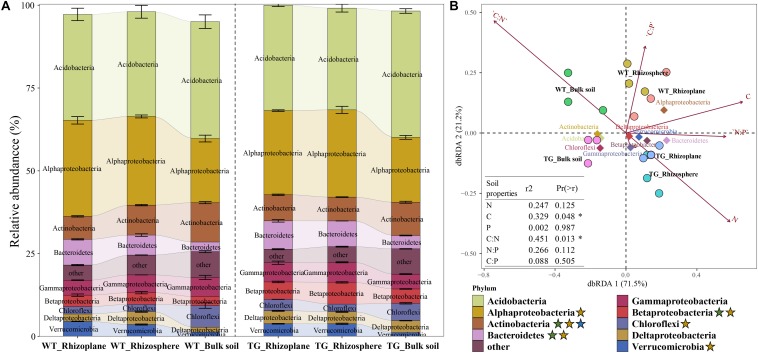
**(A)** Bacterial relative abundance with Phylum of each zone in two cultivars. The green star represents that the relative abundance of the bacteria is different between breeds; the yellow star represents that the relative abundance of the bacteria is different among different soil layers; and the blue star represents that the relative abundance of the bacteria is different both between breeds and among different soil layers. **(B)** Distance-based Redundancy analysis of different zones, abundant classes, and 6 environmental factors (arrows) indicates the dominant communities and influential environmental factors.

The relative abundance of bacterial communities based on phylum clearly differed between the TG and WT groups. An obvious fluctuation was observed between the relative abundances of the rhizoplane and rhizosphere bacterial communities in WT, whereas those of the TG plants tended to be similar. The relative abundance of Actinobacteria, Bacteroidetes, and Betaproteobacteria varied between breeds, and the relative abundance of Alphaproteobacteria, Actinobacteria, Bacteroidetes, Betaproteobacteria, Chloroflexi, and Verrucomicrobia were different among the layers. The relative abundance of Actinobacteria was different not only between breeds but also among layers (Figure 3A). The different compartments (71.5%) and sugarcane cultivars (21.2%) were observed to explain the variation in the bacterial composition in dbRDA (Figure 3B). The relationships between the main bacterial populations from the three zones and environmental factors were analyzed by dbRDA, showing that the most strongly affected populations were those of Alphaproteobacteria, Betaproteobacteria, Bacteroidetes, Deltaproteobacteria, Gammaproteobacteria, Chloroflexi, Acidobacteria, Actinobacteria, and Verrucomicrobia. Among them, Alphaproteobacteria, Bacteroidetes, Betaproteobacteria, Gammaproteobacteria, Deltaproteobacteria, and Verrucomicrobia were the predominant groups of the rhizoplane and rhizosphere areas, whereas Chloroflexi, Acidobacteria, and Actinobacteria were mainly present in the bulk soil zone. Among the environmental factors, the C/N ratio (*R*^2^ = 0.451, *P* = 0.013), TC content (*R*^2^ = 0.329, *P* = 0.048), and TN content (*R*^2^ = 0.247, *P* = 0.125) had the greatest influence on the bacterial community. In particular, the TC content was associated with Alphaproteobacteria, TN content was associated with Betaproteobacteria, Deltaproteobacteria, and Verrucomicrobia, and the C/N ratio was most strongly associated with the bulk soil area, mainly dominated by populations of Actinobacteria and Acidobacteria (arrows in Figure 3B).

### Network Analyses of Bacterial Communities Among the Three Zones

**FIGURE 4:**
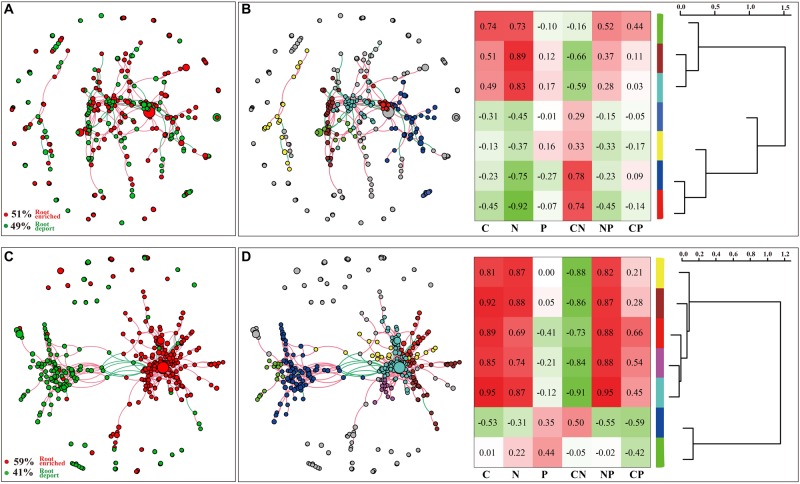
**(A,C)** DESeq2 differential abundance indicates the enrichment and deportation of OTUs within the overall area of three zones in two WT and TG, respectively. Each node represents an individual OTU, and the red edge is drawn between OTUs if they are positively correlated, while the green edge is drawn between OTUs if they are negatively correlated. **(B,D)** OTUs are divided into seven modules using the molecular ecological network analyses (MENAP), indicating the ecological network relationship between OTUs. Module-Eigen Gene analyses indicates the Module correlation with environmental factors and the Module-Eigen Gene hierarchy structure. The heatmap shows the correlation between modules and environmental factors, and the hierarchy clustering located on the right shows the Pearson correlation among module eigengenes.

**FIGURE 5:**
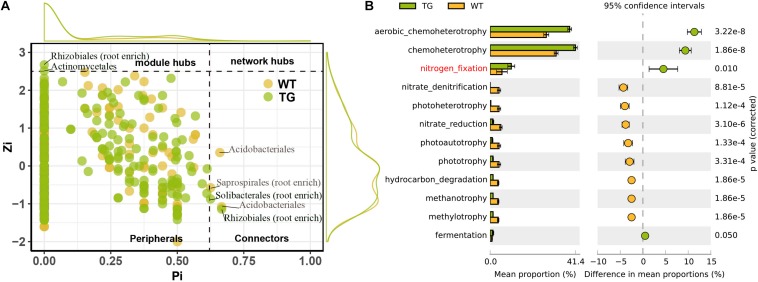
**(A)** Network module separation and modularity calculation analysis shows dominantly functioning bacterial communities. Each dot represents an OUT in two sugarcanes. The x-Zi represents within-module connectivity and the y-Pi represents among-module connectivity. **(B)** Map prokaryotic clades to established metabolic or other ecologically relevant functions based on DESeq2 shows the ecological functions of root bacterial communities in TG and WT, indicating the enhanced ecological functions of TG root bacterial communities compared with WT.

The DESeq2 differential abundance analysis showed that 59% of the OTUs of TG plants were enriched in the rhizocompartments and 41% were detected in the bulk soil area, representing a statistically significant difference from the relatively random distribution of OTUs detected in the WT areas. Moreover, there was a strong positive correlation between the OTUs at the root-associated enrichment area of TG plants (Figures 4A,C). The MENA pipeline analysis divided all the identified OTUs into seven modules, and the distribution of these modules clearly differed between the TG and WT plants. The OTUs in the five modules of the root-associated area of TG plants showed a strong correlation with environmental factors, with negligible differences between the OTUs in these five modules. In contrast, the structure of the OTUs in the five modules representing colonization in the root-associated area of TG was significantly different from those in the other two modules in the bulk soil area (Figures 4B,D). The Module-EigenGene analysis showed that the eigengenes within the TG submodules that clustered into two groups were significantly correlated. Furthermore, one of the groups comprising five submodules exhibited significantly positive correlations with the TC and TN contents and the N/P ratio (Figures 4B,D). Network module separation and modularity calculation showed that the majority of the OTUs were peripherals, with most of their links remaining within their own modules. A total of two nodes were identified as connectors in the TG plants, and these OTUs were derived from Soilbacteriales and Rhizobiales, which were both enriched in the roots. In addition, two other nodes were identified as module hubs, which were mainly derived from Rhizobiales, similar to the connectors, and Actinomycetales (Figure 5A). The Map Prokaryotic clades tool was used to associate the OTUs to established metabolic or other ecologically relevant functions for predicting the strengthened ecological functions of the bacterial communities in TG plants. This analysis showed that communities related to chemoheterotrophy and nitrogen fixation were significantly stronger in the TG plant environment than in WT environments (Figure 5B).

## Discussion

Through this field experiment, we demonstrated that drought-resistant TG sugarcane shapes the root-associated bacterial community assembly, which may in turn contribute to the ability of the host plant to respond appropriately to abiotic environmental stress. Thus, our results confirm that the diversity and composition of the bacterial communities of TG plants considerably differed from those of WT, with respect to both root-associated area and bulk soil, indicating that the genetic modification altered the plant-associated bacterial communities. The host plant genotype explained a significant portion of the variation in the diversity and composition of the bacterial communities. In addition, variation in the TG bacterial communities was more strongly correlated with soil environmental factors than that of the WT, indicating that some compounds in the root exudates have specific effects on the bacterial communities of TG plants. Finally, the rhizoplane and rhizosphere compartments of TG plants were more closely associated, whereas the dissimilarity in the bacterial communities between the rhizosphere and bulk soil was greater than that of the WT. Here, we discuss how these three main results can provide new insights into the factors that shape root-associated bacterial communities and their ecological relevance.

### Variation in Bacterial Community Diversity in TG Sugarcane

In the present study, we investigated the influence that the plant genotype exerts on bacterial community diversity in three layers (the rhizoplane, rhizosphere, and bulk soil), which is extremely correlated with the environmental factors (TC, TN, and TP contents). We found obvious variations in the root bacterial community diversity between the TG and its parental non-TG variety WT (Figures 1, 2), which have been reported by several studies in other plants (Brusetti et al., 2005; Fang et al., 2005; Li et al., 2018). In the rhizosphere, alterations in the structure, abundance, and diversity of the bacterial communities show great differences in transgenic rice and corn, which are consistent with our results (Brusetti et al., 2005; Fang et al., 2005; Li et al., 2018). However, some studies have reported conflicting results. In drought-tolerant crops, such as transgenic corn expressing the *Hahb*-*4* gene and transgenic rice expressing the *CaMSRB2* gene, only minor effects on the root-associated bacterial community were observed (Sohn et al., 2016; Ibarra et al., 2020). These conflicting results are likely due to the different genes that were modified in the plants. Hamonts et al. (2018) demonstrated that sugarcane-associated bacterial assemblage is primarily determined by plant compartment, followed by other factors such as the growing region and sugarcane variety. Indeed, plant genotype is responsible for some of the variations observed in root microbiomes, suggesting an active role of the host in the establishment of the communities (Colombo et al., 2017). Additionally, we also found a stronger correlation between the relative metrics of bacterial diversity and environmental factors in TG compared with those in WT, especially for the TC and TN contents (Figures 1C,D, 2B), suggesting that the changes in the soil environment of the root microbiome influence the bacterial community diversity. A strong relationship between root exudates and microbial diversity has been previously proven (Eisenhauer et al., 2017). Furthermore, the root exudates from GM plants strongly influence the rhizosphere microbial communities (Dunfield and Germida, 2004), and the quantity and quality of the root exudates are determined by plant genotype (Badri and Vivanco, 2009). Several studies have shown that the expression of several drought-inducible genes in an ABA-independent pathway is regulated by the DREB transcription factors (Agarwal et al., 2006; Upadhyay et al., 2017). Moreover, overexpression of *Ea-DREB2* in sugarcane leads to a higher photosynthetic rate and chlorophyll content than those of WT sugarcane under drought stress (Augustine et al., 2015). Plants may release up to 20% of their photosynthesis products into the soil, providing a basis for the establishment of plant–microorganism interactions that will benefit plant growth by, for example, increasing the availability of mineral nutrients or the production of phytohormones (el Zahar Haichar et al., 2008). Besides, a previous study showed that C cycling enzyme potential activities increased with inorganic N availability, while those of N cycling enzymes increased with C availability (Bowles et al., 2014), indicating that the increases in TC and TN in the root-associated area of TG plants might be related to the soil enzyme activities. Overall, our analysis of bacterial diversity revealed that the plant genotype is one of the primary factors contributing to changes in root bacterial diversity due to changes in the physicochemical environment of the microorganisms.

### Similar Bacterial Composition of the Rhizoplane and Rhizosphere in TG Sugarcane

In our study, we confirmed that the similar bacterial composition of the rhizoplane and rhizosphere in TG plants was related to the changes in environmental factors. The rhizoplane is the root surface where the host plants are in direct contact with the rhizosphere soil. Based on a study of the root-associated microbial community assembly, the microbial community associated with the roots was proposed to be assembled in two steps: the rhizosphere is first colonized by a subset of the bulk soil community, and then the rhizoplane and endosphere are colonized by a subset of the rhizosphere community (Sasse et al., 2018). The dynamics of microbiome acquisition in our study provide experimental support for this model, given that step 2 of the microbial community assembly is consistent with our data. That is, we observed an increase in the relative microbial abundance in the TG rhizoplane and a reduction in abundance in the rhizosphere, suggesting that some bacteria migrate from the rhizosphere to the rhizoplane (Figure 3A). It has been reported that several plant growth-promoting bacteria colonize in the rhizosphere of sugarcane under drought stress (Pereira et al., 2019). Besides, previous studies have suggested that the phylum Proteobacteria comprises several plant growth-promoting rhizobacteria (PGPR) (Bruto et al., 2014), which may facilitate plant growth by promoting the acquisition of nutritional resources such as N, P, and iron (Vurukonda et al., 2016). In our study, the dominant taxa in the root-related area were Proteobacteria, Verrucomicrobia, and Bacteroidetes (Figure 3B), especially the Proteobacteria including Betaproteobacteria, Gammaproteobacteria, and Deltaproteobacteria, which observed apparent increases in the rhizoplane of TG compared with WT plants (Figure 3A), suggesting an increase in beneficial bacterial communities. In moisture-limited soils, the relative abundance of the phyla Proteobacteria, Verrucomicrobia, and Bacteroidetes was found to decrease (Naylor and Coleman-Derr, 2017). On the contrary, Proteobacteria and Bacteroidetes were enriched in the rainy season (Barnard et al., 2013). These reports indicate the intimate relationship between the phyla mentioned above and the drought-resistant capacity of plants and also suggest the contribution made by those phyla to the enhanced drought-resistant ability of the TG plants in our study. Additionally, TC and TN were detected as the most important contributors to the variations in the bacterial communities (Figure 3B), indicating that bacterial community distribution changes with changes in environmental factors. The rhizosphere is the soil area that is most strongly influenced by the exudates released by the roots. Thus, an assembly of the rhizosphere microbiome is also influenced by the root exudates to a certain degree, which can help select beneficial soil microbial communities (Backer et al., 2018; Williams and de Vries, 2020). Therefore, the recruitment of TG rhizoplane bacteria might represent the beneficial bacterial selection from the root exudates, especially the recruitment of Proteobacteria. Indeed, exudation has been shown to play an active role in bacterial proliferation in the rhizosphere soil (Baudoin et al., 2003).

### Intimate Relationship Between the Bacterial Communities of the Rhizocompartments in TG Sugarcane

In the present study, the enhanced drought-resistant ability of TG sugarcane is closely related to the intimate relationship between the bacterial communities of the rhizocompartments in TG plants due to both the function of plant root exudation and the beneficial bacterial communities colonized in the root-related area. The rhizoplane and rhizosphere communities are extremely close, and thus these zones are commonly regarded as a continuum (Johri et al., 2003). We found a closer relationship along this continuum in the TG plants than in the WT plants (Figures 4C,D), indicating that the roots of TG plants have a more dynamic activity to uptake more nutrients from the rhizosphere soil. As mentioned above, PGPR along with nitrogen-fixing bacteria are rhizosphere organisms with well-established beneficial effects on plant growth and health (Mendes et al., 2013). In our study, Rhizobiales (Alphaproteobacteria), which belongs to PGPR (Bresson et al., 2013), was identified as a highly enriched member of the core functional bacterial community in all the three zones of TG plants, and the nitrogen-fixing and chemoheterotrophic functions of bacteria in the TG plants were stronger than those of the WT (Figures 5A,B), both of that indicating the enhanced nutrition-absorbing ability of the TG plant bacterial community. Mineral nutrients (inorganic carbon, inorganic nitrogen, and immobile phosphate) can be dissolved by the release of some compounds of the root exudates (e.g., organic acids and amino acids) that are used by rhizosphere-dwelling microbes (Song et al., 2012; Canarini et al., 2019). It has been reported that members of Alphaproteobacteria can efficiently use carbon from metabolites generated by primary assimilators in the sugarcane rhizosphere (Da Costa et al., 2018), which is consistent with our results that the Alphaproteobacteria population increased with the enhancement of TC content in the root-associated area. In addition, changes in microbial communities can act as a feedback to plant growth (Williams and de Vries, 2020). Some specific soil microbes have been confirmed to have the ability to modify the metabolite composition of the whole plant (Fernandez et al., 2012). Plant-associated microorganisms also constitute a strong sink for plant carbon, thereby increasing the concentration gradients of metabolites and affecting root exudation (Canarini et al., 2019). Our study revealed that the levels of nutrients (TC, TN, and TP) in the TG rhizosphere increased to feed more bacteria, especially beneficial communities, residing around the roots. Plant strategies for nutrient foraging may be strongly affected by the root-associated microbial population, especially the dominant beneficial communities (Pii et al., 2015). Therefore, the changing soil environment around the root may not only be the result of root exudation but also of the activity of certain beneficial bacterial populations colonized in the rhizosphere. Such an intensified and beneficial root–microbiome interaction is expected to facilitate the growth and development of the plant and further enhance the plant’s resistance to abiotic stresses.

## Conclusion

We investigated the effects of TG sugarcane harboring the drought-resistant gene *Ea-DREB2B* on the bacterial communities of the root-associated layers (rhizoplane and rhizosphere) and bulk soil. Our results support the influence of alterations in plant genotypes by genetic modifications on plant growth and health due to the feedback from changes induced in the surrounding environment. Accordingly, the diversity and composition of the bacterial community were altered by the genetic modification in sugarcane. Most importantly, we identified a stronger and more similar relationship between the rhizoplane and rhizosphere bacterial communities and a more distant relationship between the rhizosphere and bulk soil bacterial communities in TG than in WT plants, due to a change in the soil environment caused by the alteration in root exudation. The enhancement of specific ecological functions (nitrogen fixing and chemoheterotrophy) of the TG bacterial communities further indicated their stronger beneficial effects for the plant. Overall, our study provides evidence that sugarcane root-related bacterial communities can be altered by modification in the *Ea-DREB2B* gene, which influences the ABA-mediated pathway to enhance the photosynthetic rate in plants. As DREBs are important genes for crop improvement, by enhancing the resistance of plants, we focused on the effects of TG sugarcane on the bacterial communities that interact with plants. The results will help in understanding the mechanisms of drought resistance induced by DREBs. Furthermore, our study provides information about the effects of GM plants on soil bacterial communities. However, root-associated bacterial communities are influenced by numerous factors (e.g., genotype, temperature, soil texture, and soil enzymes activities), and therefore, a comprehensive evaluation of the effects of transgenic plants on bacterial communities should be conducted taking into consideration other potentially influential factors in the future.

## Data Availability Statement

The datasets generated for this study can be found in the NCBI SRP238824.

## Author Contributions

XZ and ZW contributed to design of the experiments, data analysis, and manuscript writing. YJ contributed to experimentation. QL, HY, and MZ contributed to data interpretation and manuscript preparation.

## Conflict of Interest

The authors declare that the research was conducted in the absence of any commercial or financial relationships that could be construed as a potential conflict of interest.

## Footnotes

1 http://ieg2.ou.edu/MENA/
