# Supplementary material for: Effects of Drought-Tolerant Ea-DREB2B Transgenic Sugarcane on Bacterial Communities in Soil
Source: Front Microbiol. 2020 May 5;11:704. doi: 10.3389/fmicb.2020.00704 (PMC7214759; doi:10.3389/fmicb.2020.00704)
Supplement: Supplementary file 1 [file Data_Sheet_1.PDF]

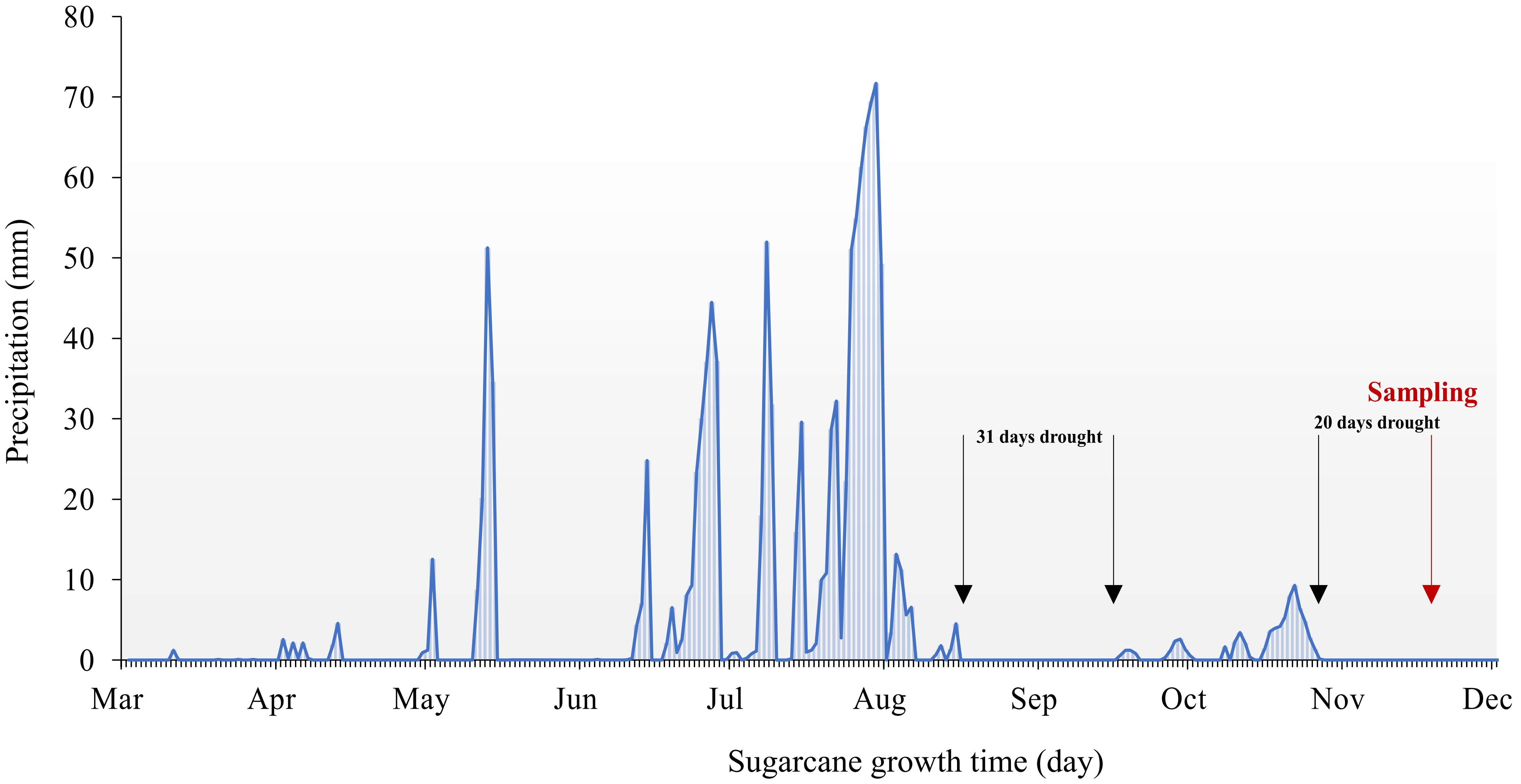

Fig S1. Map of precipitation during sugarcane growth. From August 16 to September 16, there are 31 days drought during this timescale. From September 17 to October 29, the total precipitation is 72.03mm. Before sampling, there are 20 days drought from October 30 to November 18.
